# Effect of intranasal breast milk administration on cerebral oxygenation, vital signs, and transition time to full oral feeding in preterm infants: a randomized controlled study

**DOI:** 10.1007/s00431-026-06922-6

**Published:** 2026-04-16

**Authors:** Adalet Yücel, Sibel Küçükoğlu, Murat Konak

**Affiliations:** 1 Department of Nursing, Faculty of Nursing, Selçuk University, New Istanbul Cad, Alaeddin Keykubat Campus, Akademi Mah, Selcuklu, Konya, 42250 Türkiye; 2 Department of Pediatrics, Faculty of Medicine, Selçuk University, Konya, Türkiye

**Keywords:** Breast milk, Infant, Intranasal administration, Near-infrared spectroscopy, Vital signs

## Abstract

This study examined the effects of intranasal breast milk administration on cerebral oxygenation, vital signs, and time to transition to full oral feeding in preterm infants. This assessor-blinded, randomized controlled trial included 40 preterm infants with gestational ages between 28^+0^ and 36^+6^ weeks. Infants in the intervention group received 0.2 mL of fresh breast milk intranasally three times daily for 3 days. The control group continued to receive routine clinical care. During each intervention period, cerebral oxygenation and vital signs were recorded at baseline (T0), 5 (T1), 15 (T2), and 30 (T3) minutes after the intervention. The time required for preterm infants to achieve full oral feeding was monitored until discharge. Cerebral oxygenation levels of preterm infants who received intranasal breast milk were significantly higher than the control group and respiratory rate was lower at some measurement times. No significant differences were found between groups in oxygen saturation, heart rate, time to full oral feeding, length of hospital stay, vomiting frequency and defecation frequency.

*Conclusion*: Intranasal breast milk administration has the potential to increase cerebral oxygenation in preterm infants. The maintenance of oxygen saturation and heart rate, along with good tolerance of the procedure, suggests that this intervention may be safe and feasible.

*Trial registration*: The trial was registered on ClinicalTrials.gov on 21 May 2024 (Identifier: NCT06706115).

**Table:** 

**What is Known:** • *Intranasal breast milk administration has been reported to have therapeutic potential in improving brain injury in preterm infants.* • *Intranasal administration of breast milk is known not to cause significant side effects in preterm infants diagnosed with intraventricular hemorrhage.*	
**What is New:** • *Intranasal breast milk administration may increase cerebral rSO₂ in preterm infants.* • *Intranasal breast milk administration did not affect preterm infants’ heart rate, SpO₂ levels, time to transition to full oral feeding, length of hospital stay, daily vomiting, and stooling frequency.*

**Supplementary Information:**

The online version contains supplementary material available at 10.1007/s00431-026-06922-6.

## Introduction

Studies conducted in animal models and adults have demonstrated that the intranasal route serves as an alternative pathway for the administration of stem cells, growth factors, or pharmacological agents [1–4]. It is suggested that the intranasal route, with its rich vascular density, provides a biological pathway that enables therapeutic agents to be delivered directly to the central nervous system via the olfactory epithelium and trigeminal nerve [5, 6]. This route is considered a noninvasive, easily applicable method that can bypass the blood–brain barrier for the delivery of therapeutic agents to the central nervous system and it is suitable for repeated administrations [1, 4, 7]. In recent years, studies evaluating the feasibility of intranasal administration of breast milk and its potential effects have been reported [8, 9]. It is thought that following intranasal administration of breast milk, the stem cells and other bioactive components it contains are transported to brain tissue via the permeability of the nasal epithelium [10].

Intranasal administration of breast milk in preterm infants is a relatively novel approach and most existing studies have primarily focused on its effects on brain injury, intraventricular hemorrhage (IVH) and neurodevelopmental outcomes [8, 9, 11, 12]. Furthermore, the intervention has been reported not to cause any significant adverse reactions in preterm infants diagnosed with IVH, and the infants were able to tolerate the procedure [9]. However, it is known that even routine care procedures in preterm infants can affect cerebral circulation and physiological stability [13, 14]. Therefore, there is a need for a better understanding of the effects of interventions performed in neonatal intensive care units (NICUs) on the cerebral and hemodynamic stability of preterm infants [15]. In this context, further studies are needed to better understand the effects of intranasal breast milk administration in preterm infants [9].

Given that the intranasal route may facilitate the delivery of substances via the olfactory epithelium [4] and that the application site involves the nasopharyngeal region, the intervention has the potential to produce effects similar to those previously reported for breast milk odor exposure [16, 17] and oropharyngeal breast milk administration [18]. However, in the literature, studies on intranasal breast milk administration are frequently conducted in preterm infants diagnosed with IVH. Due to its richness in stem cells and unique benefits, breast milk may potentially exhibit protective or therapeutic effects when administered intranasally, not only for infants diagnosed with IVH but also for other preterm infants. Therefore, studies are needed to better understand the effects of intranasal breast milk in different sample groups. However, no randomized controlled trial has been found investigating the effects of intranasal breast milk administration in preterm infants on cerebral oxygenation (rSO_2_), vital signs, or the time to transition to full oral feeding.

The aim of this study was to determine the effect of intranasal breast milk administered to preterm infants on their cerebral rSO_2_ levels, vital signs and the time to transition to full oral feeding. The study also investigated the effect of the intervention on the infants’ daily vomiting and defecation frequency and length of hospital stay. The study hypotheses were as follows: preterm infants receiving intranasal breast milk would differ from the control group in terms of **(H**_**1a**_**)** cerebral rSO_2_, **(H**_**1b**_**)** vital signs (heart rate, oxygen saturation-SpO₂, respiratory rate), **(H**_**1c**_**)** time to achieve full oral feeding, **(H**_**1d**_**)** vomiting frequency, **(H**_**1e**_**)** defecation frequency, and **(H**_**1f**_**)** length of hospital stay.

## Methods

### Study design and setting

This study was an assessor-blinded, randomized controlled trial. The study was conducted between June 2024 and October 2025 in the NICU of a university hospital, involving preterm infants. The NICU is equipped with a hospital-grade breast pump, which allows mothers to express milk on-site if they wish. Additionally, mothers can express milk at home and deliver it to the NICU feeding nurse.

### Participants and eligibility criteria

The study sample consisted of 40 preterm infants (20 intervention, 20 control). The study included infants who were (i) between 28 and 37 weeks gestational age, (ii) had a birth weight greater than 1000 g, (iii) were fed enterally, (iv) had an Apgar score greater than 7, and (v) had available breast milk. It was planned that infants would be withdrawn from the study if breast milk became unavailable during the study period, if the infant’s clinical condition deteriorated (e.g., initiation of respiratory support or diagnosis of infection), if the infant achieved full oral feeding after the start of the study, or if the parents requested withdrawal from the study. However, no such circumstances occurred during the study period.

### Sample size

As no similar reference study could be found with comparable outcomes during the planned study period, the a priori sample size calculation was performed using the G*Power 3.1.9.6. programme, with reference to the transition period data for full oral feeding from the study by Fu et al. [18]. In the power analysis for the *t*-test, using a two-tailed hypothesis, an effect size of 1.22, 95% power, a significance level of 0.05, and an allocation ratio of 1:1, it was determined that a total of 38 preterm infants (intervention = 19, control = 19) would provide an adequate sample size. Anticipating potential data losses, it was planned to include 20 infants in each group. As a result, 20 infants were included in each group, thus obtaining a total sample size of 40.

### Randomization and blinding

In the study, allocation of infants to the study groups was performed using stratified randomization combined with block randomization methods [19]. Infants were stratified into two groups according to gestational age (28–32⁺⁶ and 33–36⁺⁶ weeks). Subsequently, 10 blocks were created, each containing 4 participants and including codes A and B. Each code was placed in a sealed, opaque envelope in sequence. Which group the codes A and B corresponded to was determined by a sealed envelope lottery method (A = Intervention, B = Control). All these procedures were carried out by an independent researcher not involved in the study. For each infant meeting the inclusion criteria, the investigator conducting the study opened the envelope to determine the group assignment immediately before the intervention. As the intervention was administered by the researchers, researchers’ blinding was not feasible. Study data were analyzed by a statistician not involved in this study, thereby ensuring statistical blinding. The study was conducted and reported in accordance with the Consolidated Standards of Reporting Trials (CONSORT) guidelines [20] (Supplementary File 1).

### Data collection tools

Data were collected using three structured forms developed by researchers in line with the literature [8]. The “Infant Information Form” included clinical characteristics of preterm infants such as gender and date of birth. The “Physiological Parameter Follow-up Form” was used to record cerebral rSO₂ and vital signs throughout the intervention period. The “Nutrition Follow-up Form” documented feeding-related data during the same observation period, including number of vomiting and defecation frequency, time to achieve full oral feeding and length of hospital stay. Cerebral rSO₂ levels and vital signs were measured using the “INVOS™ 5100CNear-Infrared Spectroscopy (NIRS)(Medtronic,Minneapolis,MN,USA)” monitor, “Masimo Pulse Oximeter” and “Philips Patient Monitor (Philips Medical Systems, Orlando, FL, USA)” which is routinely used for clinical monitoring in the NICU where the study was conducted.

### Outcome measures

**Fig. 1:**
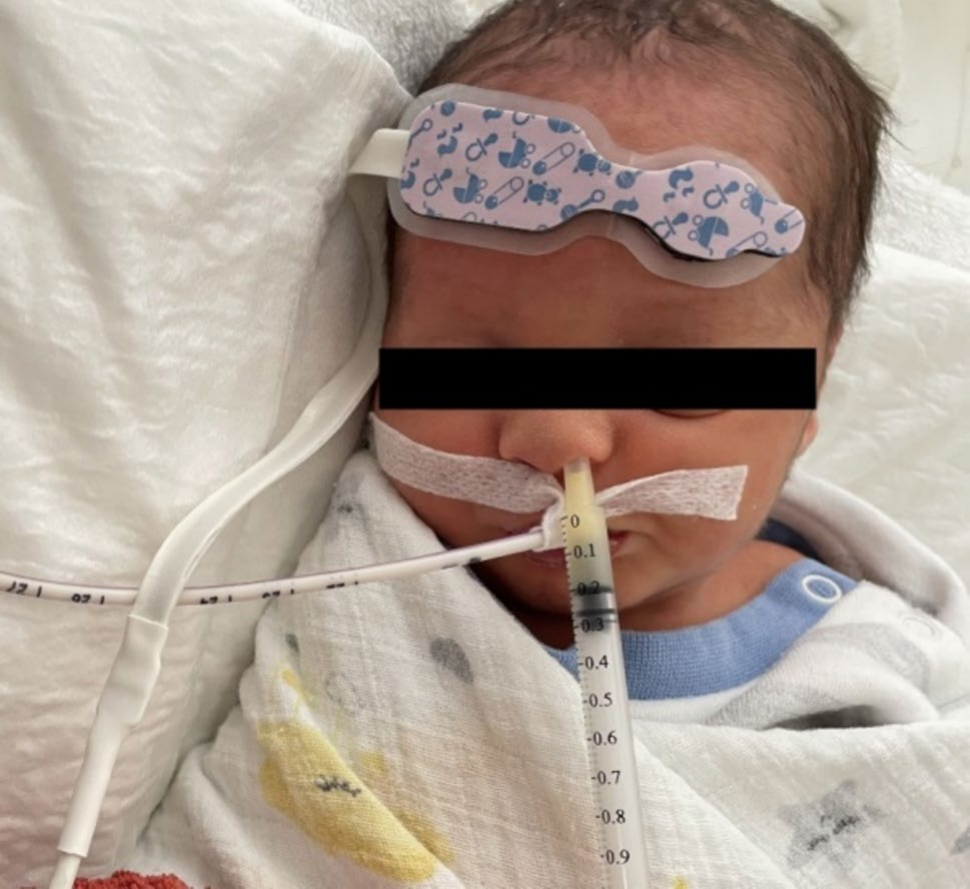
Intranasal breast milk administration to the intervention group in the study

Parents of infants eligible for inclusion were informed about the scope of the study and written informed consent was obtained. Descriptive data recorded in the “Infant Information Form” were retrieved from medical records. The NIRS sensor was placed on the infant’s forehead by a nurse prior to the intervention (Fig. 1). Cerebral rSO₂ and vital signs were recorded at four time points during three daily intervention periods: before the intervention (T0) and at 5 (T1), 15 (T2), and 30 (T3) minutes after the intervention. The same measurement time points were applied to the control group. However, infants in this group received only routine clinical care. Data for the “Nutrition Follow-up Form” were recorded daily from medical records.

### Procedure

The study procedures were conducted in accordance with the previously published study protocol [21]. Fresh breast milk was obtained from mothers for use in the intervention. Fresh breast milk was stored according to recommendations reported in the literature [22]. For the intervention, 0.2 mL of breast milk was prepared in a 1-mL syringe and administered to the intervention group at room temperature. A pilot study was conducted with two preterm infants to evaluate the study protocol prior to the data collection process. No changes were made to the study procedure following the pilot study, and the data obtained from these infants were not included in the study.

## Intervention group

According to the literature, intranasal administration of 0.2 mL of breast milk has been reported to be well tolerated by preterm infants [9]. Accordingly, in the present study, preterm infants in the intervention group received 0.2 mL of fresh breast milk via the intranasal route per administration. The intervention was performed three times daily for three consecutive days. Immediately before the intervention, baseline data (T0) recorded and the researcher then administered 0.2 mL of fresh breast milk at room temperature, instilling 0.1 mL into the right nostril and 0.1 mL into the left nostril (Fig. 1). Apart from the intervention, all nursing interventions in this group (feeding, care etc.) were continued in accordance with routine clinical practice.

## Control group

In the control group, no intervention was administered and routine clinical care was maintained. To ensure comparability in the duration of the data collection process, measurements were obtained and recorded at time points corresponding to those used in the intervention group.

### Data analysis

Data were analyzed using IBM SPSS Statistics version 26 (IBM Corp., Armonk, NY, USA). Descriptive statistics were reported as frequencies (*n*), means, standard deviations, percentages, and interquartile ranges. Normality was assessed based on skewness values within ± 2.0 and kurtosis values below 7.0, and the data were determined to be normally distributed [23]. Comparisons of continuous variables between groups were performed using the independent sample *t*-test, while categorical variables were analyzed using Pearson’s chi-square test or Fisher’s exact test, as appropriate. To examine time and group effects, a mixed-design analysis of variance (ANOVA) was applied. Effect sizes were interpreted according to Cohen’s classification [24]. Statistical significance was set at *p* < 0.05.

### Ethics

Ethical committee approval for the study was obtained from the Non-Interventional Clinical Research Ethics Committee of the Faculty of Nursing at Selçuk University (7 February 2024, decision no: 2024/11). Written informed consent was obtained from the parents of preterm infants who met the inclusion criteria. The study was conducted as part of a doctoral thesis. This study was supported by the Health Institutes of Türkiye Group A3 Emergency R&D Doctoral/Medical Specialty Students Project Support Program (project number 43294). The study protocol was registered on ClinicalTrials.gov on 21 May 2024 (Identifier: NCT06706115).

## Results

When the descriptive characteristics of the infants were compared between the study groups, all parameters showed similar distributions across groups, indicating homogeneity in baseline characteristics (*p* > 0.05, Table 1).

**Table 1:** Comparison of descriptive characteristics of infants by groups (*n* = 40)

Parameter	Groups		*p*-value
	Intervention (*n* = 20)	Control (*n* = 20)	
Gender	*n* (*%*)	*n* (*%*)	0.752
Girl	9 (45)	10 (50)	
Boy	11 (55)	10 (50)	
Type of birth	*n* (*%*)	*n* (*%*)	0.999
Cesarean section	15 (75)	15 (75)	
Vaginal delivery	5 (25)	5 (25)	
Apgar score (5 min)			0.759
*X* ± *SD*	8.30 ± 0.47	8.25 ± 0.55	
*M* (*IQR*)	8 (1)	8 (0)	
Gestational age (weeks)			0.717
*X* ± *SD*	32.60 ± 1.47	32.80 ± 1.96	
*M* (*IQR*)	33 (2)	33 (2)	
Postnatal age (weeks)			0.065
*X* ± *SD*	34.10 ± 1.12	34.70 ± 0.86	
*M* (*IQR*)	34 (1)	35 (1)	
Birth Weight (grams)			0.817
*X* ± *SD*	1897.00 ± 374.52	1930.00 ± 508.62	
*M* (*IQR*)	1910 (674)	1855 (801)	
Weight on the first day of study (grams)			0.249
*X* ± *SD*	1953.50 ± 278.59	2074.25 ± 368.04	
*M* (*IQR*)	1993 (423)	2085 (478)	

Data are presented as mean (X) ± SD or median (IQR), as appropriate

**Fig. 2:**
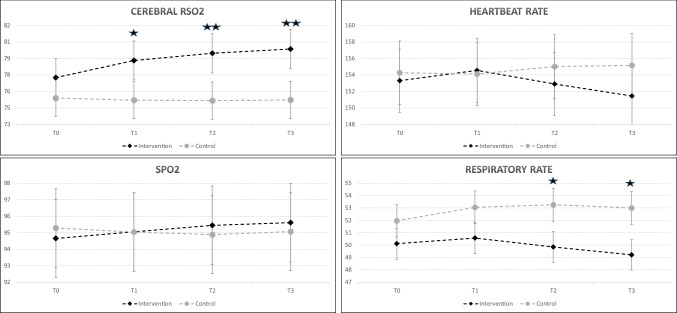
Changes in cerebral rSO₂, heartbeat rate, SpO₂, and respiratory rate over time in intervention and control groups. Data are presented as mean ± SD. *p < 0.05, **p < 0.01 for between-group comparisons at each time point

No statistically significant differences were observed between groups in the mean cerebral rSO₂ levels at the baseline measurement (*p* > 0.05). However, at T1, T2, and T3 measurement times cerebral rSO₂ levels in the intervention group were significantly higher than those in the control group (*p* < 0.05). Mean cerebral rSO₂ values were 78.82 ± 3.71 vs. 75.20 ± 5.07 at T1, 79.47 ± 3.62 vs. 75.17 ± 4.92 at T2 and 79.86 ± 3.48 vs. 75.22 ± 4.93 at T3 in the intervention and control groups, respectively. Cerebral rSO₂ levels showed a significant group × time interaction, with values in the intervention group showing greater change than in the control group (*p* < 0.001, Fig. 2).

There was no statistically significant difference in the mean SpO_2_ values of preterm infants between the groups (*p* > 0.05). Additionally, a significant group × time interaction effect was identified, indicating greater changes in mean SpO₂ levels in the intervention group compared to the control group (*p* < 0.001, Fig. 2).

No statistically significant differences were observed between groups in mean heart rate (*p* > 0.05). The group × time interaction analysis revealed that mean heart rate exhibited greater changes in the intervention group compared to the control group (*p* = 0.020, Fig. 2).

When comparing the mean respiratory rates of infants between groups, while there was no statistically significant difference in the mean T0 and T1 measurements (*p* > 0.05), the mean respiratory rates at T2 and T3 measurement times were statistically lower in the intervention group than in the control group (*p* < 0.05). Mean respiratory rate values were 49.84 ± 4.92 vs. 53.26 ± 4.84 at T2 and 49.21 ± 5.03 vs. 53.00 ± 5.08 at T3 in the intervention and control groups, respectively. It was determined that the group × time interaction of mean respiratory rates was significant, and mean respiratory rates showed more variation in the intervention group than in the control group (*p* < 0.001, Fig. 2).

The mean time to full oral feeding and length of hospital stay were similar between the intervention and control groups, with no statistically significant differences observed (*p* > 0.05, Table 2). No statistically significant differences were observed between groups, within groups, or for the group × time interaction in terms of vomiting and defecation frequency (*p* > 0.05, Table 2).

**Table 2:** Comparison of the transition time to full oral feeding, length of hospital stay, vomiting and defecation frequency measurements of infants by groups (*n* = 40)

	Group		*p*-value
	Intervention (*n* = 20)	Control (*n* = 20)	
Transition time to full oral feeding (*day)*			0.125
*X* ± *SD*	18.15 ± 7.49	23.20 ± 12.27	
*M* (*IQR*)	16 (7)	19 (11)	
Length of hospital stay (*day*)			0.404
*X* ± *SD*	21.70 ± 7.31	24.40 ± 12.30	
*M* (*IQR*)	20 (10)	20 (11)	
Vomiting frequency	***X* ± *SD***	***X* ± *SD***	
*1. Day*	0.35 ± 0.67	0.75 ± 1.07	0.165
*2. Day*	0.15 ± 0.37	0.60 ± 1.05	0.077
*3. Day*	0.15 ± 0.37	0.25 ± 0.44	0.442
Defecation frequency			
*1. Day*	3.95 ± 1.23	3.75 ± 0.97	0.572
*2. Day*	3.85 ± 1.14	3.50 ± 1.15	0.339
*3. Day*	3.50 ± 1.15	3.55 ± 1.05	0.886

Data are presented as mean (X) ± SD or median (IQR), as appropriate

## Discussion

To our knowledge, this study is the first to investigate the effect of intranasal breast milk administration on cerebral rSO_2_ levels. The findings demonstrated that preterm infants receiving intranasal breast milk had significantly higher mean cerebral rSO₂ levels compared to the control group. It is known that substances administered intranasally reach the central nervous system via the olfactory epithelium and trigeminal nerve [3, 4, 7]. It has been reported that exposure to maternal breast milk odor or maternal nipple odor can increase cerebral rSO₂ levels in preterm infants [25, 26]. In this context, intranasal breast milk administration is considered to promote cortical activation by increasing cerebral rSO₂ levels in a manner comparable to exposure to breast milk odor. Studies in animal models have demonstrated the neuromodulatory effects of breast milk on neurogenic tissue [27]. Therefore, it seems likely that intranasal administration of breast milk increased cerebral rSO₂ levels not only through olfactory stimulation but also through its biochemical components.

The study found that the mean SpO₂ and heart rate of preterm infants administered intranasal breast milk were similar to those of the control group, but the mean respiratory rate was lower than that of the control group in some measurements after the intervention. In the literature, the only study examining the physiological effects of intranasal breast milk administration in preterm infants was conducted by Hoban et al. [9]. In their study, it was determined that intranasal administration of 0.2 mL of breast milk twice daily for 28 days did not cause any serious adverse reactions in preterm infants diagnosed with intraventricular hemorrhage, but a total of 32 reactions occurred, 30 of which were minor, which were most likely related to the intervention. However, it has been noted that recorded reactions such as increased ventilator settings may be related to the infant being extubated or to concomitant clinical conditions such as pneumoperitoneum [9]. The study by Hoban et al. [9] differs from the present study in terms of intervention frequency, monitoring schedule and sample characteristics. While the study by Hoban et al. [9] was conducted with infants diagnosed with IVH, almost all of whom received positive pressure respiratory support, the present study involved relatively healthier infants who did not require any respiratory support. Therefore, it is considered important that this study findings reveal the effect of intranasal breast milk on the vital signs of preterm infants, as independently as possible from the influence of additional pathological variables. Intranasal breast milk is also known to be used to reduce nasal congestion and provide nasal moistening [28]. Therefore, it is plausible that, in the intervention group, enhancement of nasal patency may have contributed to within-group improvements in vital signs.

In this study, the transition time to full oral feeding, length of hospital stay, daily frequencies of vomiting and defecation in preterm infants receiving intranasal breast milk were found to be similar to those in the control group. Only one study was found that reported the effect of intranasal breast milk on the feeding and discharge time of preterm infants [8]. Demir et al. [8] reported that infants administered intranasal breast milk had similar transition to full enteral feeding and hospital stay durations compared to infants in the control group. In the literature, it has been reported that the breast milk odor and the administration of breast milk to the oropharyngeal area potentially may shorten the transition to full oral feeding and reduce hospital stay [18, 29]. It has also been shown that the breast milk odor can reduce the frequency of vomiting and increase the frequency of defecation [17]. In this study, the transition period to full oral feeding, the length of hospital stay, and the daily frequency of vomiting and defecation did not differ between groups, which may be related to the frequency and duration of the intervention.

## Limitations

First, in this study, the effects of intranasal administration of breast milk were evaluated when the intervention was applied three times per day over a period of 3 days. Due to the lack of long-term follow-up data, it was not possible to determine whether the observed increase in cerebral rSO₂ levels was sustained over time, whether it translated into neurodevelopmental outcomes, or whether vital stability was maintained after completion of monitoring. Second, the absence of monitoring for medical treatments such as caffeine, which can affect vital signs in preterm infants, is considered a limitation. Finally, the inability to perform researcher-blinded testing due to the intervention and data collection being done by the researcher is another limitation.

## Conclusion

In this study, intranasal administration of breast milk in preterm infants was found to increase cerebral rSO₂ levels compared with the control group and to reduce mean respiratory rates at certain measurement time points. The study also found that preterm infants who received intranasal breast milk had similar heart rate, SpO₂ levels, the transition time to full oral feeding, length of hospital stay, daily frequencies of vomiting, and defecation to those in the control group. The findings of this study suggest that intranasal breast milk administration may be a potential intervention to increase cerebral rSO₂ in preterm infants. The similarity in heart rate and SpO₂ levels between infants who received intranasal breast milk and the control group indicates that the infants tolerated the intervention and that intranasal breast milk may be a safe approach in terms of physiological stability. However, further studies investigating different intervention frequencies and durations, as well as long-term outcomes, are needed to more clearly define the safety and efficacy profile of this intervention.

## Supplementary Information

Below is the link to the electronic supplementary material.

ESM1 (DOCX 222 KB)

ESM2 (DOCX 108 KB)

## Data Availability

The data that support the findings of this study are available on request from the corresponding author.

## Abbreviations

CONSORT: Consolidated standards of reporting trials
IVH: Intraventricular hemorrhage
NICU: Neonatal intensive care unit
NIRS: Near-infrared spectroscopy
rSO₂: Oxygenation
SpO₂: Oxygen saturation

## Declarations

### AI declaration statement

The authors declare that no artificial intelligence tools were used in the preparation of this manuscript.

### Clinical trial registration

The study protocol was registered on ClinicalTrials.gov on 21 May 2024 (NCT06706115). The protocol of this study was previously published in Nursing in Critical Care. The present manuscript reports the final results obtained based on this protocol. Yücel A, Küçükoğlu S, Konak M, 2026. The Effect of Intranasal Breast Milk Administration on Cerebral Oxygenation, Vital Signs and Time to Full Oral Feeding in Preterm Infants: a Randomised Controlled Study Protocol. Nursing in Critical Care, 31, 1, e70262. 10.1111/nicc.70262.

## References

[1] Hanson LR, Frey WH (2008) Intranasal delivery bypasses the blood-brain barrier to target therapeutic agents to the central nervous system and treat neurodegenerative disease. BMC Neurosci 9(3):1–4 19091002 10.1186/1471-2202-9-S3-S5 PMC2604883

[2] Scafidi J, Hammond TR, Scafidi S, Ritter J, Jablonska B, Roncal M et al (2014) Intranasal epidermal growth factor treatment rescues neonatal brain injury. Nature 506(7487):230–4 24390343 10.1038/nature12880 PMC4106485

[3] Ying W (2008) The nose may help the brain: intranasal drug delivery for treating neurological diseases. Future Neurol 3(1):1–4

[4] Woensel M, Wauthoz N, Rosière R, Amighi K, Mathieu V, Lefranc F et al (2013) Formulations for intranasal delivery of pharmacological agents to combat brain disease: a new opportunity to tackle GBM? Cancers 5(3):1020–1048 24202332 10.3390/cancers5031020 PMC3795377

[5] Li Y, Wu H, Jiang X, Dong Y, Zheng J, Gao J (2022) New idea to promote the clinical applications of stem cells or their extracellular vesicles in central nervous system disorders: combining with intranasal delivery. Acta Pharm Sin B 12(8):3215–3232 35967290 10.1016/j.apsb.2022.04.001 PMC9366301

[6] Shen W, You T, Xu W, Xie Y, Wang Y, Cui M (2023) Rapid and widespread distribution of intranasal small extracellular vesicles derived from mesenchymal stem cells throughout the brain potentially via the perivascular pathway. Pharmaceutics 15(11):2578 38004556 10.3390/pharmaceutics15112578 PMC10675165

[7] Zhang Y, He K-J, Zhang J-B, Ma Q-H, Wang F, Liu C-F (2021) Advances in intranasal application of stem cells in the treatment of central nervous system diseases. Stem Cell Res Ther 12(1):210 33762014 10.1186/s13287-021-02274-0 PMC7992869

[8] Demir GS, Ozdemir OM, Turgut M, Pekal Y, Koyuncu E, Güngör O et al (2025) Impact of intranasal administration of fresh breast milk in very low birth weight infants with germinal matrix-intraventricular hemorrhage. Cureus. 10.7759/cureus.80416 40213722 10.7759/cureus.80416 PMC11984797

[9] Hoban R, Gallipoli A, Signorile M, Mander P, Gauthier-Fisher A, Librach C et al (2024) Feasibility of intranasal human milk as stem cell therapy in preterm infants with intraventricular hemorrhage. J Perinatol 44(11):1–6 38688998 10.1038/s41372-024-01982-8

[10] Malhotra A (2023) Neurotherapeutic potential of intranasal administration of human breast milk. Pediatr Res 94(6):1–2 10.1038/s41390-023-02759-z PMC10665176 37495680

[11] Keller KF, Oberthuer A, Schafmeyer L, Mehler K, Kuhr K et al (2019) Intranasal breast milk for premature infants with severe intraventricular hemorrhage-an observation. Eur J Pediatr 178(2):199–206 30386923 10.1007/s00431-018-3279-7 PMC6339661

[12] Gallipoli A, Unger S, El Shahed A, Fan C-P, Signorile M, Wilson D et al (2025) Outcomes after intranasal human milk therapy in preterm infants with intraventricular hemorrhage. J Perinatol 45(2):202–7 39384614 10.1038/s41372-024-02147-3

[13] Dix LML, Shepherd K, Polglase GR, Miller SL, Sehgal A, Wong FY (2020) The cerebral hemodynamic response to pain in preterm infants with fetal growth restriction. Front Pediatr 8:268 32537447 10.3389/fped.2020.00268 PMC7267032

[14] Altimier L, Phillips R (2016) The neonatal integrative developmental care model: advanced clinical applications of the seven core measures for neuroprotective family-centered developmental care. Newborn Infant Nurs Rev 16(4):230–244

[15] Limperopoulos C, Gauvreau KK, O’Leary H, Moore M, Bassan H, Eichenwald EC et al (2008) Cerebral hemodynamic changes during intensive care of preterm infants. Pediatrics 122(5):e1006–e1013 18931348 10.1542/peds.2008-0768 PMC2665182

[16] Yu L, Tao Y, Jia P, Li L, Lv T, Wang L et al (2025) Effect of breast milk olfactory experience on physiological indicators in very low birth weight infants: a randomized clinical trial. Sci Rep 15(1):20590 40596001 10.1038/s41598-025-05809-0 PMC12217873

[17] Yücel A, Küçükoğlu S, Soylu H (2024) The effect of breast milk odor on feeding cues, transition time to oral feeding, and abdominal perfusion in premature newborns: a randomised controlled trial. Biol Res Nurs 26(1):160–175 37682253 10.1177/10998004231200784

[18] Fu ZY, Huang C, Lei L, Chen LC, Wei LJ, Zhou J et al (2023) The effect of oropharyngeal colostrum administration on the clinical outcomes of premature infants: a meta-analysis. Int J Nurs Stud 144:104527 37295286 10.1016/j.ijnurstu.2023.104527

[19] Kim J, Shin W (2014) How to do random allocation (randomization). Clin Orthop Surg 6(1):103 24605197 10.4055/cios.2014.6.1.103 PMC3942596

[20] Hopewell S, Chan A-W, Collins GS, Hróbjartsson A, Moher D, Schulz KF et al (2025) CONSORT 2025 statement: updated guideline for reporting randomised trials. Lancet 405(10489):1633–40 10.1016/S0140-6736(25)00672-5 40245901

[21] Yücel A, Küçükoğlu S, Konak M (2026) The effect of intranasal breast milk administration on cerebral oxygenation, vital signs and time to full oral feeding in preterm infants: a randomised controlled study protocol. Nurs Crit Care 31(1):e70262 41542903 10.1111/nicc.70262

[22] American Academy of Pediatrics (2025) Milk storage guidelines 2025 Available from: https://www.aap.org/en/patient-care/breastfeeding/milk-storage-guidelines/?srsltid=AfmBOorReLfgByY2AKcrMXEt4VXiGuIKMEzOq4VC2h3ovShcathsaLuw.

[23] Kim HY (2013) Statistical notes for clinical researchers: assessing normal distribution (2) using skewness and kurtosis. Restor Dent Endod 38(1):52–54 23495371 10.5395/rde.2013.38.1.52 PMC3591587

[24] Cohen J (1988) Statistical power analysis for the behavioral sciences, 2nd ed. Academic, New York, NY

[25] Di Battista C, Grometto A, Strozzi M, D’Adamo E, Lapergola G, Maconi A et al (2025) Short-long term near infrared spectroscopy patterns after different milk regimens olfactory stimuli in late preterms. Ital J Pediatr 51(1):113 40221740 10.1186/s13052-025-01912-0 PMC11994012

[26] Frie J, Bartocci M, Kuhn P (2020) Neonatal cortical perceptions of maternal breast odours: a fNIRS study. Acta Paediatr 109(7):1330–1337 31782829 10.1111/apa.15114

[27] Kaps J, Georgieva VS, Oberholz L, Kribs A, Brachvogel B, Keller T (2023) Human preterm colostrum stimulates outgrowth in neurogenic tissue. Pediatr Res 94(6):1906–1910 37433903 10.1038/s41390-023-02721-z PMC10665184

[28] Keller T, Körber F, Oberthuer A, Schafmeyer L, Mehler K, Kuhr K et al (2019) Intranasal breast milk for premature infants with severe intraventricular hemorrhage-an observation. Eur J Pediatr 178(2):199–206 30386923 10.1007/s00431-018-3279-7 PMC6339661

[29] Yildiz A, Arikan D, Gözüm S, Taştekın A, Budancamanak İ (2011) The effect of the odor of breast milk on the time needed for transition from gavage to total oral feeding in preterm infants. J Nurs Scholarsh 43(3):265–273 21884372 10.1111/j.1547-5069.2011.01410.x

