# Supplementary figures and images for: Effect of intranasal breast milk administration on cerebral oxygenation, vital signs, and transition time to full oral feeding in preterm infants: a randomized controlled study
Source: Eur J Pediatr. 2026 Apr 16;185(5):272. doi: 10.1007/s00431-026-06922-6 (PMC13083323; doi:10.1007/s00431-026-06922-6)

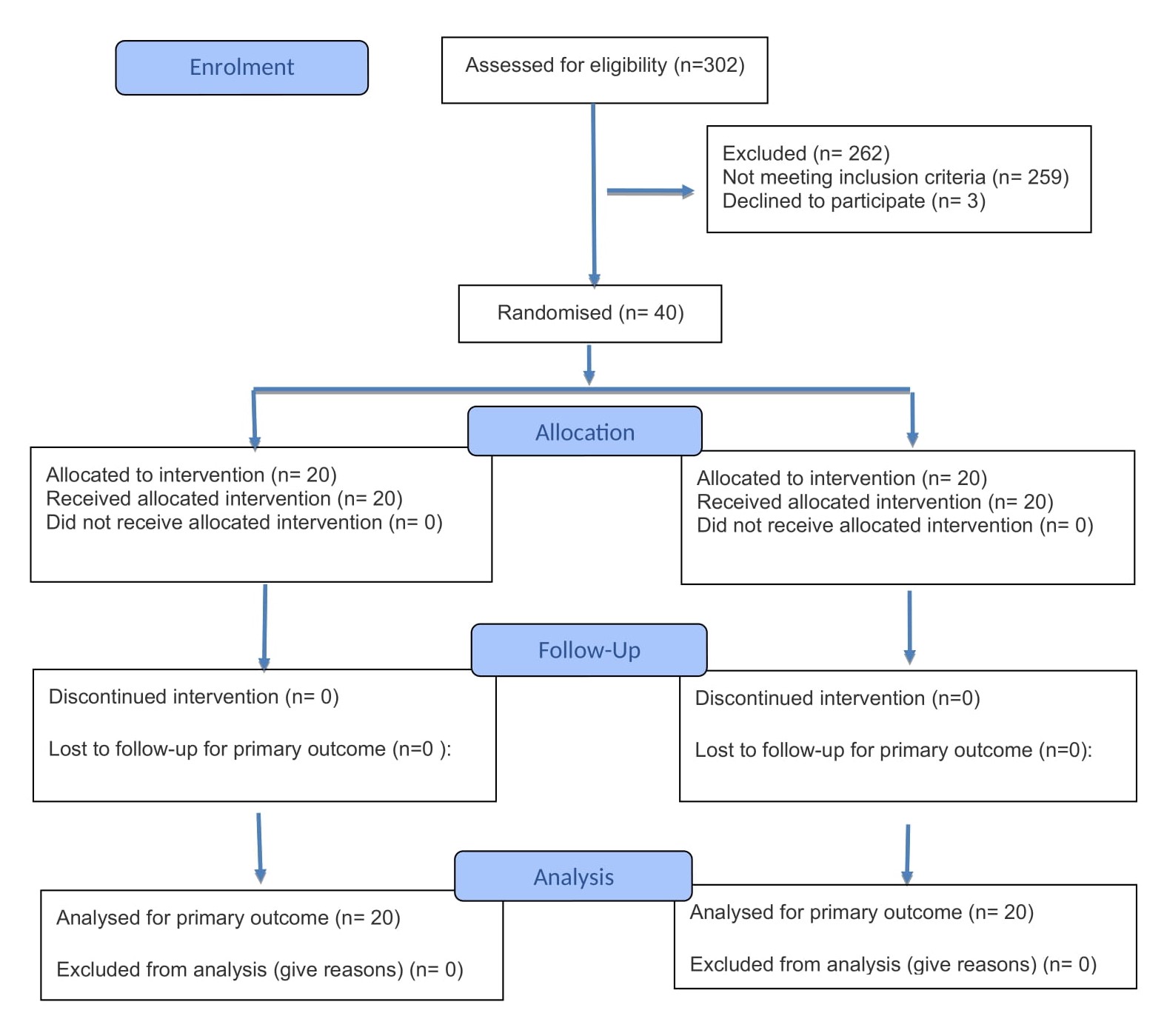


**Supplementary File 1.** CONSORT Flow Diagram of the Study.

Supplement: Supplementary file 1 — (DOCX 222 KB) [file 431_2026_6922_MOESM1_ESM.docx]
